# Collective Dynamics Underlying Allosteric Transitions in Hemoglobin

**DOI:** 10.1371/journal.pcbi.1003232

**Published:** 2013-09-19

**Authors:** Martin D. Vesper, Bert L. de Groot

**Affiliations:** Department of Theoretical and Computational Biophysics, Max Planck Institute for Biophysical Chemistry, Göttingen, Germany; UNC Charlotte, United States of America

## Abstract

Hemoglobin is the prototypic allosteric protein. Still, its molecular allosteric mechanism is not fully understood. To elucidate the mechanism of cooperativity on an atomistic level, we developed a novel computational technique to analyse the coupling of tertiary and quaternary motions. From Molecular Dynamics simulations showing spontaneous quaternary transitions, we separated the transition trajectories into two orthogonal sets of motions: one consisting of intra-chain motions only (referred to as *tertiary-only*) and one consisting of global inter-chain motions only (referred to as *quaternary-only*). The two underlying subspaces are orthogonal by construction and their direct sum is the space of full motions. Using Functional Mode Analysis, we were able to identify a collective coordinate within the tertiary-only subspace that is correlated to the most dominant motion within the quaternary-only motions, hence providing direct insight into the allosteric coupling mechanism between tertiary and quaternary conformation changes. This coupling-motion is substantially different from tertiary structure changes between the crystallographic structures of the T- and R-state. We found that hemoglobin's allosteric mechanism of communication between subunits is equally based on hydrogen bonds and steric interactions. In addition, we were able to affect the T-to-R transition rates by choosing different histidine protonation states, thereby providing a possible atomistic explanation for the Bohr effect.

## Author Summary

Hemoglobin transports oxygen from our lungs to other tissues. Its effectiveness in binding and unbinding of oxygen is based on a type of regulation called allostery. Thereby, already bound oxygen molecules in either of the four binding sites of hemoglobin increase the likelihood of oxygen binding in the other sites; the sites act cooperatively. It is known that the four protein chains of hemoglobin – each containing one oxygen binding site – need to rearrange globally or the cooperativity is lost. But how do the individual chains communicate with each other in this rearrangement? This is still not resolved on the molecular level. By applying the computational technique of molecular dynamics simulations we were able to simulate the motions of the individual atoms of hemoglobin during such a global rearrangement. We present a novel method that allows to directly analyse the coupling of motions within and between the four protein chains and thereby reveal the allosteric mechanism. That allows us to classify which amino acids are important for the cooperativity and in what way: by either pushing other amino acids away or attracting them. Also, we have observed in simulations how pH differences could affect the cooperativity.

## Introduction

Hemoglobin (Hb) consists of two 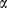 and two 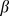 protein chains that bind oxygen to a heme group for the transport through the blood. These binding sites act cooperatively, i.e. the binding affinity for 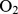 in one site is increased after 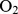 binding in one of the other sites. The remarkable efficiency of Hb's cooperative oxygen binding gave rise to extensive experimental and theoretical work [1]–[9].

Today, many high-resolution structures are available, including oxy states, deoxy states, carbon monoxide bound structures and structures of a large number of point mutations [10]. Dioxygen dissociation curves are recorded for many of the mutants, making it possible to see the effect of individual residues on the cooperativity. Dynamical information is obtained from e.g. spectroscopic studies, observing transition states in the oxy to deoxy transition, and analysing specific bonds during CO dissociation [11].

In addition to experiments, Molecular Dynamics (MD) simulations have provided insight for shorter timescales from 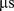 down to ps while maintaining the full atomistic picture of Hb. The work of Shadrina et al. and of Lepeshkevich et al. focused on 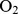 diffusion in Hb studied with MD [3], [4]. Ramadas and Rifkind simulated conformational changes due to perturbations of the heme pocket for methemoglobin dimers [5]. In the work by Mouawad et al., the Hb T-to-R transition was enforced by restraining the Hb coordinates with decreasing structural distance to the R-state structure [6]. The study from Yusuff et al. focused on 100 ns simulations from different crystallographic structure models [9].

Recently, Hub and co-workers observed for the first time spontaneous reproducible transitions from the T- to the R-state during MD simulations [7]. They described a tendency for the 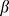-chains to couple more strongly to the quaternary motion than the 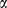-chains.

In the present study we investigated how the local intra-chain motions couple to the global inter-chain motions on a molecular level. To this end, we enhanced the statistical basis to 21 transition trajectories, and developed a method which allows to characterize the coupling between global and local motions. For this purpose, we first separated global from local motions and then identified the coupling mechanism between them. We analysed the resulting coupling collective coordinate on the level of molecular contacts, shedding light on the molecular allosteric mechanism of hemoglobin. In addition, by using a different set of histidine protonations to mimick simulations at different pH, we were able to show a reduction in the number of transition trajectories. This constitutes a possible explanation for the Bohr effect in Hb [12], [13].

## Results

### Molecular Dynamics Simulations

From the Hb simulations carried out by Hub and co-workers [7], the ones starting from the T-state with doubly protonated and thus positively charged 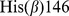 (all other histidines neutral) showed transitions to the R-state in all three runs. In our study, we extended these simulations to improve the statistical basis of the T-to-R transitions. From a total of 50 simulations (200 ns long each; 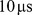 total simulation time) 22 showed a spontaneous transition. For the further steps, only transition trajectories were taken into account.

### Coupling of Quaternary and Tertiary Motions

**Figure 1:**
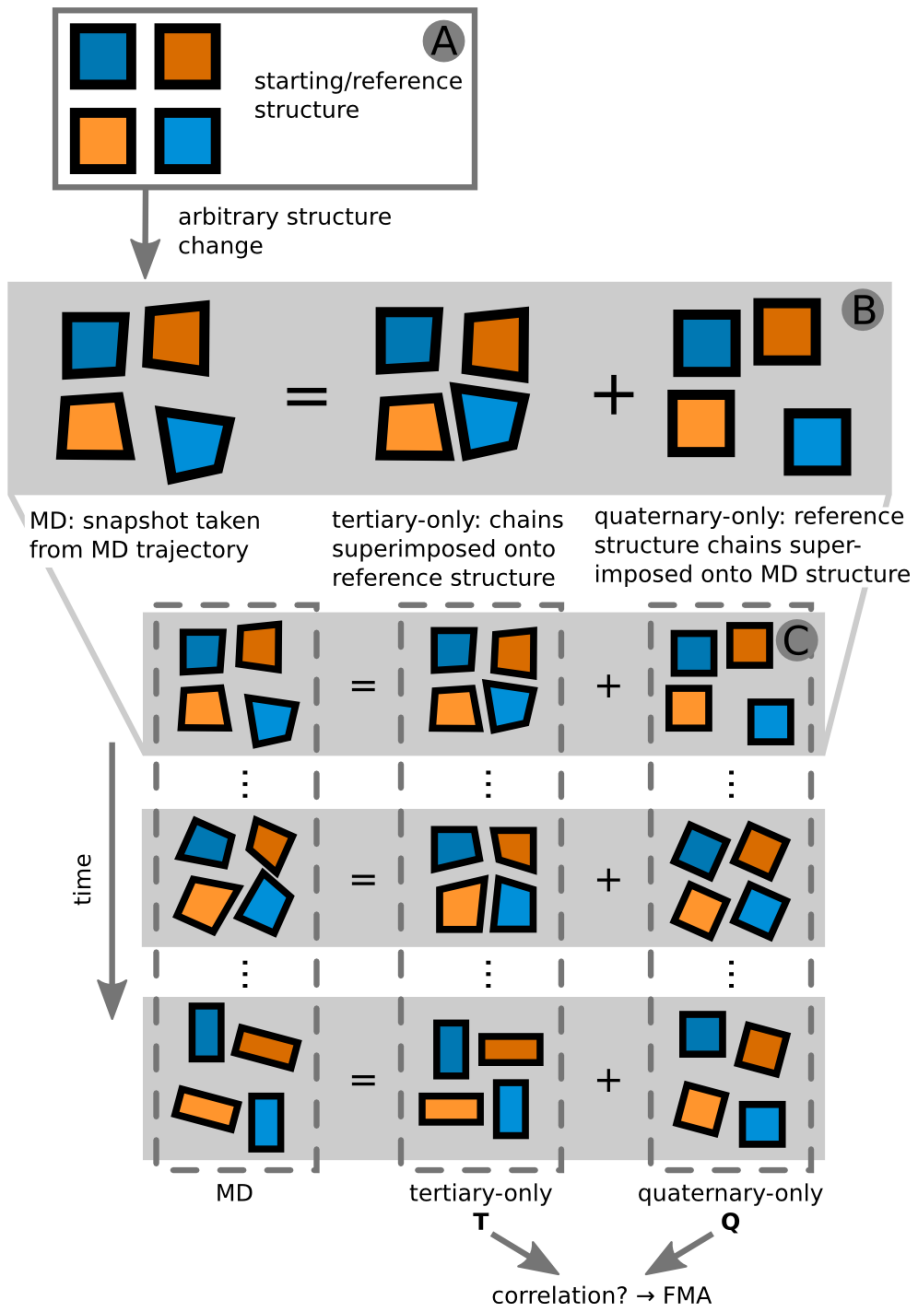
Illustration of the separation procedure of the MD trajectories into tertiary-only and quaternary-only trajectories. At the top it is shown how a single MD snapshot is decomposed (B) with respect to the reference structure (A). This procedure is applied to all snapshots yielding the two desired trajectories of tertiary-only and quaternary-only motions (C). The schematic system was chosen to resemble Hb with its four chains.

Starting from our T-to-R transition trajectories, to analyse the interplay of local and global motions, we separated local from global motions as the first step. Here, the MD transition trajectories were decomposed into two trajectories: quaternary-only (Q) and tertiary-only (T). The first consists of inter-chain motions with the Hb chains translating and rotating as rigid bodies and the second contains intra-chain motions, omitting the global movements. The combination of the Q and T trajectories yields the full MD trajectories. For a visual explanation of the basic idea of the decomposition see Figure 1, for a detailed description see *Materials and Methods*. The two corresponding subspaces for Q and T are orthogonal by construction, but the actual motions inside them may still be correlated, thus reflecting the underlying allosteric mechanism in Hb. We therefore investigated if there was a coupling between local (T) and global (Q) motions. In other words: can we construct a linear combination of the T coordinates that is correlated to the Q motion?

**Figure 2:**
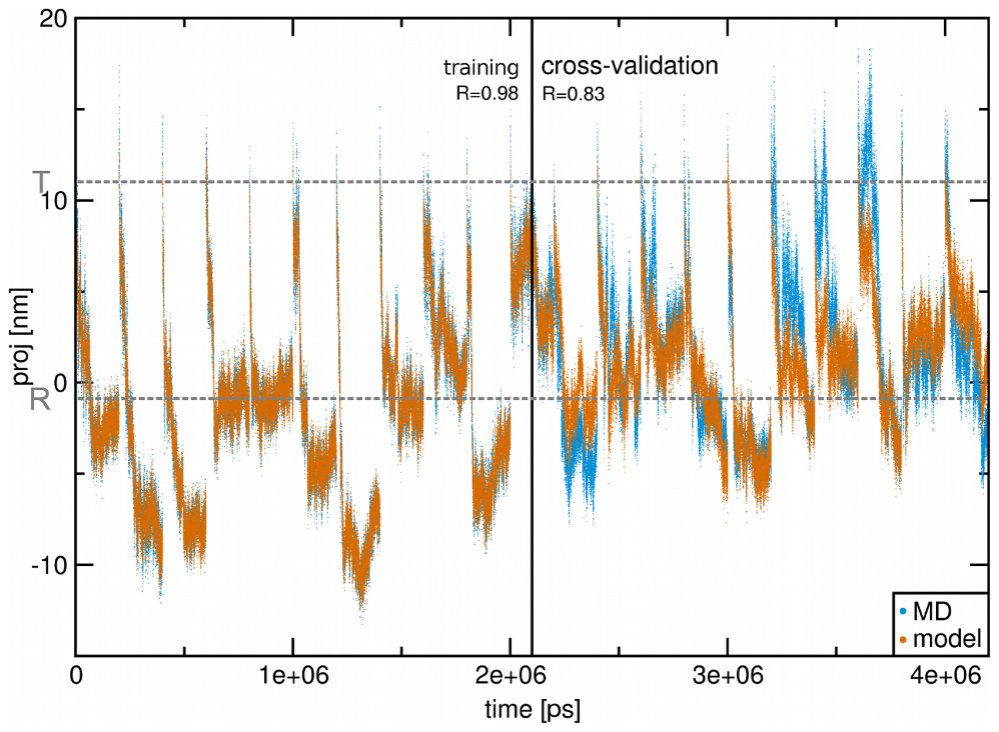
Functional Mode Analysis input data and fit results. Projections of the concatenated MD trajectories onto cQ (blue) and onto the constructed model cT (orange) are shown. The first half of the data has been used for constructing the model and the second half for cross-validation. Pearson correlation coefficients comparing MD data and FMA model for both parts are shown on top. The x-axis is the consecutive simulation time and the y-axis the projection onto the principal quaternary eigenvector in nm. The projections for the T- and R-state X-ray structures are marked in grey.

We simplified the Q trajectory by only considering the first eigenvector of a Principal Component Analysis (PCA) as the most dominant motion (referred to as cQ). For obtaining a collective coordinate within T that maximally correlates to cQ, we applied Functional Mode Analysis ((FMA), [14]) based on Partial Least Squares [15]. In our case the projection on cQ was the functional property and T the coordinates that were used as a basis to cQ from. We assessed the risk of overfitting, arising from the high dimensionality of the T space by cross-validation. The resulting tertiary FMA model correlated to the cQ motion with a Pearson correlation coefficient of 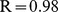 (see Figure 2). This means that despite the orthogonal nature of the two underlying subspaces, T and Q, we found a collective coordinate (named cT) within T that is strongly coupled to Q. This allows us to predict the quaternary state from the internal subunit coordinates alone, thereby providing insight into the allosteric mechanism of subunit communication through quaternary conformation changes. The cT coordinate is not similar to the tertiary difference vector of the T- and R-state – the scalar product of the two motions is 0.01 –, and thus yields novel information based on the dynamics during quaternary transitions.

Within the FMA framework it can be desirable to reweigh the individual latent vectors with their contribution to the overall variance (see [14]): While the FMA mode cT is the maximally correlated motion, which may actually be restricted, the ensemble-weighted mode cTew is the most probable motion that correlates with the functional property. For further analysis, we used this ensemble-weighted motion (cTew).

### Molecular Coupling Mechanism

**Figure 3:**
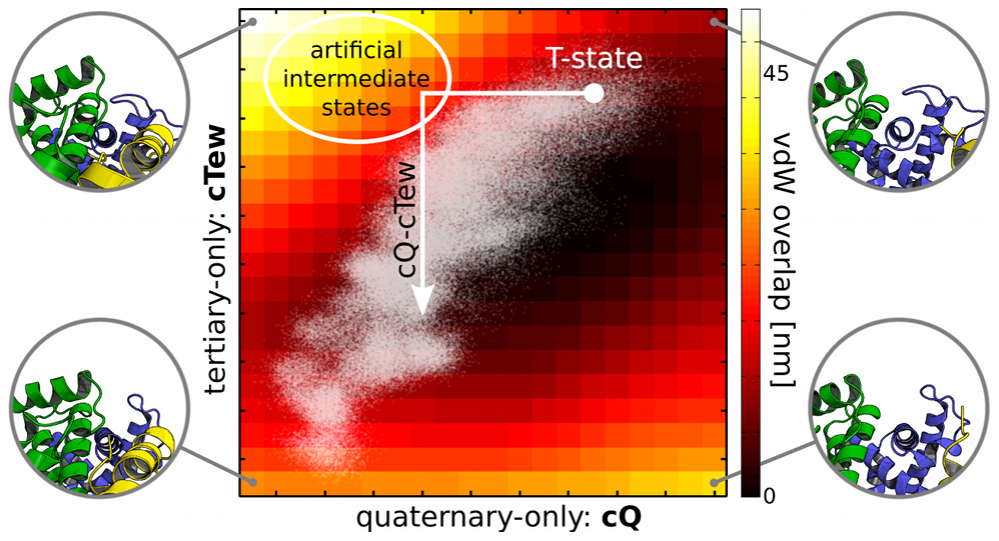
Graphical representation of the vdW overlap analysis. Overlaps were calculated for structures in the plane spanned by cQ (x-axis) and cTew (y-axis). For the extreme structures in the four corners a zoomed-in part on the N-terminal region of the 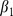-chain (green), the 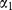-chain (blue) and the 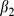-chain (yellow) of Hb is shown to illustrate the motions. Projections of the original simulation data onto this plane are shown as white dots.

To elucidate the mechanism of coupling between T and Q, we focused on the hyperplane spanned by T and Q. In this plane we chose a specific path starting from the T-state and moving first parallel to cQ and then parallel to cTew. The path is marked in white in Figure 3 and will be referred to as cQ-cTew. It artificially separates motions that are occurring simultaneously in the simulations. This allows us to classify the contacts according to their decomposition into global and local motions.

In order for the information of a local conformation to flow from one protein chain to another, it has to cross the corresponding interface. It is therefore of interest to investigate interactions at the subunit interfaces. We focused on inter-chain van der Waals overlaps and general distance based contacts to investigate the interactions underlying the allosteric coupling mechanism.

### Van der Waals Overlaps

To estimate the influence of interatomic repulsion due to steric interactions as a driving force for the allosteric coupling, we calculated the overlap of atomic van der Waals (vdW) spheres. We did this for structures in the plane spanned by cQ and cTew, and took into account only inter-chain overlaps. (Note that these structure are projected onto this plane and hence differ from the actual MD structures.) The higher the overlap in a specific structure, the more energetically unfavourable it is. As can be seen in Figure 3, the overlaps are minimal along the main diagonal while increasing when moving orthogonally. Projecting the structures from the MD simulations (white dots) onto this plane shows that they coincide with the low vdW overlap region.

### Contact Analysis

For a broader view including also attractive interactions, we monitoredinter-chain atom pairs showing a distance smaller than 3 nm. This analysis was carried out along cQ-cTew which allows us to classify the contacts according to when the contact is or is not formed along this specific path. For specific interaction types following contact patterns are expected. The two residues in contact are either:

- *pulling* at each other, breaking the contact when in the off-diagonal intermediate artificial states (see Figure 3) and maintaining it close to the T-and R-state,
- *pushing* each other, getting close only in the off-diagonal intermediate artificial states when not moving along cQ and cTew together, or
- *switching* one of the interacting residues for another while moving along cQ-cTew.

**Figure 4:**
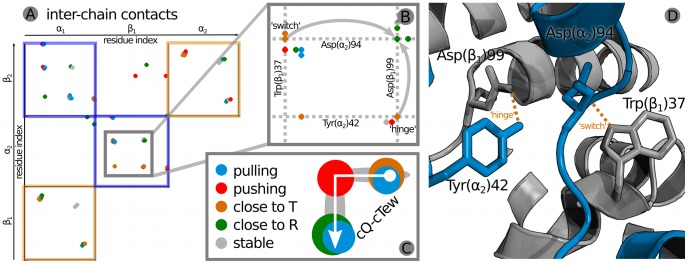
Inter-chain contact analysis. (A) The matrix of observed contacts pairs along cQ-cTew is shown. The colour of the dot indicates when along cQ-cTew the two residues are in contact and thereby defines the contact class. (B) An exemplary close-up on a contact region, which allowed us to identify contacts that are part of the ‘switch’ and ‘hinge’ region (structure shown in (D)) as measured by Balakrishnan et al. [11]. The arrows mark contacts of the 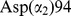 and the 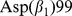 residues in which one contact partner switches when going from T-state (orange) to the R-state (green). (C) Schematic representation of the contact classifications along cQ-cTew.

Figure 4 depicts the contact analysis and classification at the example of the contact cluster around 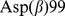.

### Pulling Contacts

In Table 1 all observed contacts are listed that fall in the first category. These contacts only stay intact if the system moves along cQ and cTew together, but break if moving in one or the other direction independently. This is the expected behaviour for contacts which must remain intact for the allosteric mechanism to function. Exemplarily, this was observed for 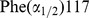 and 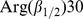. The hydrogen bond between the carboxylic oxygen of Phe and the side chain of Arg breaks while moving from the T-state towards the off-diagonal intermediate artificial states (see Figure 3), and forms again when approaching the R-state.

**Table 1:** List of observed contacts of pulling, pushing and switching type.

pulling		pushing		switching		
residue 1	residue 2	residue 1	residue 2	Residue	contact in T	contact in R
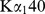	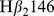	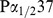	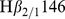	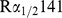	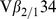	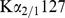
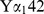	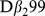	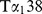	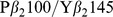	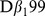 †	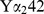	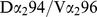
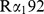	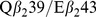	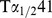	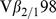	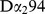 ‡	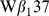	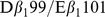
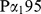	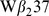	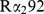	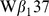	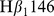	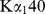	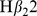
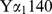	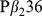	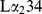	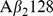			
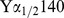	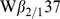	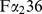	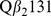			
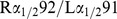	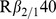	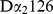	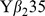			
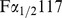	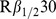	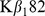	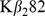			
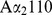	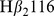	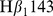	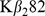			

† In Hb Kempsey the mutation 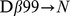 increases the 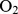 affinity [31], [32]. The hydrogen bond 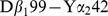 was analysed by Balakrishnan et al. and named “switch contact” [11].

‡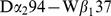 is the “hinge contact” analysed by Balakrishnan et al. Both hydrogen bonds are reported to form during transition from R to T.

### Pushing Contacts

Contacts of the second category, which appear only while moving along cQ and cTew individually, are listed in Table 1. One scenario how these contacts could be leading to the allosteric mechanism is the residues getting too close when moving only along cQ or cTew. This could be the case for vdW overlaps or repulsive coulomb interactions.

A clear example for this is the interaction of 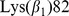 and 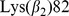 we observed: Close to the T-state both side chains are pointing into the solvent. While moving along cQ, the two 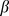 chains approach each other and bring both positively charged side chains unfavourably close. The motion along cTew relaxes this repulsive interaction by bending the N-terminal ends of the F helices (the helix notation goes back to Watson, Kendrew and Perutz [16]). Experimental studies introduced cross-links between the two lysines [17], [18]. The derived structure was described to be an intermediate between T- and R-state with characteristics of both states but no cooperativity. This is in accord with our analysis, from which we saw that a linker between the lysines would make the F helix bending impossible.

### Switching Contacts

If during the transition one residue switches an interaction partner, we expect to see the first contact disappearing and a contact with the new residue appearing. This was observed e.g. for the C-terminal 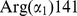. Its side chain interacts with the carboxyl group of 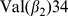, and switches along cQ-cTew so that a salt-bridge is formed between the Arg terminus and the side chain of 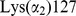. This event also has been seen in the symmetry-related counterpart independently. Further contacts of this type are listed in Table 1.

### Influence of Histidine Protonation

We aimed to analyse the effect of the histidine protonation states on the transition probabilities to elucidate the possible pH-dependence underlying the Bohr effect. For this purpose, a second set of protonation states was simulated. We chose to use the protonation states as reported by Kovalevsky et al., who used neutron protein crystallography to measure the protonation of histidine residues in the T-state [19]. Both histidine protonation states are listed in Table 2 with the Kovalevsky protonation states corresponding to a lower pH. In the case of the protonations used by Hub et al., 13 out of 20 simulations showed a transition, while in the case of the protonation state described by Kovalevsky et al., only 4 out of 20 simulations did (see Table 3). This suggests a clear dependence of the transitions on the histidine protonation state.

**Table 2:** Comparison of used histidine protonation states.

	Hub	Kovalevsky			Hub	Kovalevsky	
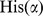	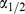	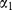 †	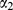	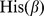	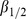	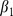	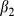
20	0‡	+1§	0	2	0	(0)$	(0)
45	0	0	0	63	0	+1	0
50	0	0	+1	77	0	0	0
58	0	+1	0	92..Fe	H	H	H
72	0	+1	+1	97	0	+1	+1
87..Fe	H*	H	H	116	0	+1	+1
89	0	0	+1	117	0	(0)	0
103	0	+1	+1	143	0	0	+1
112	0	+1	+1	146	+1	+1	0
122	0	0	0				

† The measured protonations by Kovalevsky et al. are different for the both *α* resp. *β* subunits whereas Hub et al. used symmetric protonation states.

‡ indicates the neutral side chain.

§ doubly protonated and thereby positively charged side chain.

$ Residues for which the protonation was not derived are shown in brackets. In that case we used the protonation by Hub et al.

* Histidine bound to heme groups.

**Table 3:** Simulation setup.

rvdw [nm]*	His prot.§	nr. of sims	nr. of transitions
1.0	Hub et al.	10	5
1.4	Hub et al.	20	13/12$
1.4	Kovalevsky et al.	20	4

Differences in parameters for the individual MD simulations carried out in this study.

* Lennard-Jones cut-off.

§ histidine protonation states.

$ In this case 13 transitions were observed but only 12 trajectories were included in the analysis (see *Materials and Methods*).

## Discussion

### Coupling of Quaternary and Tertiary Motions

We present a novel allosteric mechanism coupling quaternary and tertiary transitions in Hb. One fundamental component of the applied approach is a strict separation of local/tertiary and global/quaternary degrees of freedom. This guarantees that any observed coupling between both subspaces is not due to linear dependence of the respective basis vectors, but represents a true feature of the allosteric mechanism. The suggested separation algorithm is not limited to hemoglobin and can be applied to other systems with multiple chains. Also, the algorithm can be used for any definition of domains in the broader sense to separate the motions within the domains and between the domains.

The second component of the presented approach, Functional Mode Analysis, allowed us to find a linear coupling coordinate between tertiary and quaternary motions and thereby identify the allosteric coupling. Applied to hemoglobin, FMA revealed a remarkable correlation between collective coordinates from quaternary-only and tertiary-only motions. The tertiary-only coupling mode is markedly different from the tertiary structure differences between the known crystallographic R- and T-states. Thus, this mode could not have been derived solely based on the X-ray structures, but yields novel information directly based on transition trajectories between the T- and R-state. For a direct comparison of the collective coordinates cQ, cT, cTew and the crystallographic T-R difference vector (and its quaternary-only and tertiary-only component), scalar products between the coordinates are summarized in Table 4.

**Table 4:** Mutual scalar products of specific collective coordinates.

	cQ	cT	cTew	T-R full	T-R tertiary	T-R quaternary
cQ	1.00					
cT	0.01	1.00				
cTew	0.02	0.42	1.00			
T-R full	0.63*	0.01	0.03	1.00		
T-R tertiary	0.03	0.03†	0.02	0.52	1.00	
T-R quaternary	0.75*	0.00	0.01	0.84	0.02	1.00

Scalar products between the normalized vectors along collective coordinates including cQ, cT, cTew. For a comparison with the X-ray structures we also decomposed the difference vector between the T- and R-state (T-R full) in the same way it was done with the MD trajectories yielding T-R tertiary and T-R quaternary.

* The high scalar product between cQ and the crystallographic T-R difference vector (and its quaternary-only component) shows a significant similarity of the first eigenvector of the quaternary-only coordinates is similar to the crystallographic T-to-R transition.

† The small scalar product between the obtained FMA solution cT and the tertiary-only component for the crystallographic T-R difference vector indicates that cT could not have been derived solely from the T- and R-state structures.

The correlation of 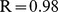 for the model training (and 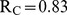 for the cross-validation) between the quaternary mode and the detected tertiary mode is high, and allows us to predict Hb's quaternary conformation for a given tertiary conformation with high accuracy. We were able to detect this coupling despite the fact that we did not take the full Q motions into account, but rather reduced the motions to the first PCA eigenvector cQ. In a future study, a more complete interaction picture may be derived by coupling the tertiary motions to the full 18 dimensional quaternary subspace. The high correlation between cQ and cT despite the near-zero overlap between the modes (scalar product of 0.01) clearly indicates towards the strong coupling between tertiary and quaternary motions as basis for the cooperativity in Hb.

The model that we used to describe the coupling of quaternary and tertiary motions is of linear nature. On the one hand, since there is no necessity for a linear coupling, non-linear models could be more suitable. On the other hand, the fact that we found a linear model with an substantial correlation in the cross-validation makes us confident that already a major part of the coupling can be described linearly. We investigated only instantaneous cQ to T coupling, although in reality there might be a lag-time due to the time required for the signal to pass. Our results, however, show that the coupling can already be identified from an analysis of instantaneous correlations. Future work may include non-linear models as well as delayed responses for the coupling, especially for systems in which the order of events is known.

### Van der Waals Overlap & Contact Analysis

An analysis of the identified coupling mode focused on the protein chain interfaces revealed key interaction residues. We suggest that the allosteric coupling between local and global motions in Hb consists of an interplay of repulsive and attractive interactions at the subunit interfaces on a similar scale.

The interaction picture we derived from our vdW overlap analysis suggests that vdW overlaps are a global driving force for the allosteric coupling. The fact that MD trajectories projected onto the plane spanned by cQ and cTew coincide with the region around the diagonal in Figure 3 that corresponds to low vdW overlap strongly points at the underlying coupling mechanism: With cTew derived to optimize the coupling between local and global motions, it also shows a strong coupling of steric repulsions between local and global motions.

Nevertheless, efforts to break down the global interactions to individual repulsive contact pairs did not yield a conclusive picture. This suggests that the vdW overlap at Hb's inter-chain interfaces does not act on a residue level, but on a broader, collective scale.

The contact analysis along cQ-cTew allowed us to classify contacts according to their behaviour along this path. By picking this path in the plane spanned by cQ and cTew, we ensure that the observed contacts are important for the coupling. If any contact pair did not play a role in the allosteric coupling, it would not have been part of the coupling of global and local motions. For a number of contacts we observed also the symmetry-related residue-pairs (if not a contact of the same type, at least the contact itself), which further indicates the significance of these contact pairs. The similar number of pulling and pushing interface interactions suggests that both types contribute to the allosteric coupling on a similar scale. The fact that steric repulsions and hydrogen bonds could not be unambiguously traced down to a residue level individually, but could in the generalized contact analysis, points at an interplay of repulsive and attractive interactions.

### Hb Montefiore and 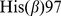


In our analysis the two residues 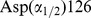 and 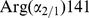 stay in contact along the full cQ-cTew path, that is close to R and T as well as in the off-diagonal intermediate artificial states. Hence, since the contact is not changing in the coupling space spanned by cQ and cTew, this salt-bridge does not seem to play a role in the coupling of local and global motions. Nevertheless, it was shown in Hb Montefiore that the mutation of 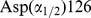 to a Tyr breaks down the cooperativity [20]. Also, while the contact is present in the crystallographic T-state structure, it is broken by an outward flip of the Aspartates in the R-state. Further studies are needed to investigate why this contact pair did not show up in our analysis. The applied dimensionality reduction from Q to cQ may have caused this. Simulations of the Tyr mutant may reveal the underlying mechanism.

Further, 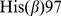 has been shown to play an important role during the quaternary transition [6], [21]. In our analysis this residue is in contact with 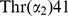, but behaved similarly to the 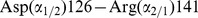 contact and stayed intact along the cQ-cTew path. The neighbouring switch region residues 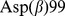 and 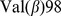 were, however, identified as ‘switching’ and ‘pushing’ contacts, respectively, confirming the crucial role of the switch region in the transition.

### Suggested Mutations

In this study, we assigned different roles in the coupling mechanism (like forming hydrogen bonds or repulsion due to van der Waals overlap) to individual amino acids. Mutagenic studies of these amino acids provide a direct means to validate the predicted role of individual amino acids in the allosteric mechanism. The observed contacts which are caused by van der Waals overlap may be reduced by mutating to residues with smaller side chain sizes. In the case of charged side chains introducing an additional charge of the same sign may increase the repulsion. Contacts including hydrogen bonds can be suppressed by using unfavourable mutations.

We suggest mutations affecting the “hinge” and “switch” contacts [11] in Table 1. Our observations extended this region by interaction partners in the R-state allowing to choose mutations affecting either the T- or R-state. By introducing a hydrogen bond donor the mutation 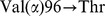 may stabilize the R-state. The central role of the 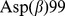 in this area makes it an interesting mutation site since it might separate the two timescales associated with the “hinge” and “switch” contacts as described by Balakrishnan et al. [11]. To explore the 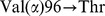 mutant we performed five simulations at 200 ns with this mutant and observed two quaternary transitions. Even though we originally expected this mutation to affect the transition rates, the fraction of transition trajectories does not significantly differ from our original simulations. A closer look at these preliminary simulations indicates that an additional hydrogen bond is indeed formed but not inter-subunit but rather with 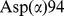 within the same 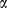 chain and may thereby only contribute a minor effect to the transition rates.

The repulsive interaction of the two 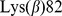 can be explored by either a mutation to Arg or even by switching both charges by a mutation to Glu, keeping the repulsive Coulomb interaction.

Further, we suggest a mutation of the 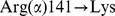 to analyse the details of this salt-bridge by this conservative mutation, leaving the charges untouched and only changing the side chain length.

### Influence of Protonation

In our simulations, we were able to lower the T-to-R transition probability by mimicking a pH shift, thereby providing a molecular picture of the Bohr effect. Future mutational studies may verify our predicted interactions and consolidate the molecular interaction mechanism of hemoglobin's allosteric coupling.

The measured histidine protonations in the T-state by Kovalevsky et al. [19] showed a large number of doubly protonated and thus positively charged side chains. By applying these protonations to our MD simulations we were able to observe significantly lowered transition probabilities from the T- to R-state as compared to the original setup with singly protonated histidines. This is consitent with the Bohr effect: a preference for the T-state at lower pH.

Even though our simulations are not long enough to have reached equilibrium, our observations can be taken as a proof of concept of the electrostatic interactions stabilizing the T-state and thereby underlying the pH-driven Bohr effect. During the T-to-R transition residues at the 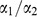 and the 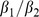 interfaces get closer. Positively charged histidine residues at these interfaces add a repulsive Coulomb force, rendering the transition energetically more unfavourable. Interestingly, most of the histidines that are changed from neutral to positive when applying the T-state protonations (see Table 2), are located on the outside of Hb and not at chain interfaces. This might point at long-range interactions complementing the short-range interface interactions. A careful introduction of additional histidines by mutation – as pH sensitive switches – may increase the Bohr effect. Also, calculation of free energy differences between the T- and R-state upon protonating histidine residues may yield direct, quantative insight into the contribution of individual histidines to the total Bohr effect.

### Force-Field Dependence

To estimate if our choice of force-field influences the transition process, we carried out further simulations using the CHARMM27 force-field [22]. Three of those are using the TIP3P water model and the other three the CHARMM water model TIPS3P. Within 60 ns, we see that all three simulations with TIP3P and two out of three TIPS3P simulations showed a quaternary T-to-R transition. In addition to that, in their recent work Yusuff et al. also see quaternary transitions using the AMBER-99SB force field [23] when starting from the T-state in two simulations at 100 ns [9]. Both results show that the spontaneous 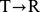 transitions are reproducible across force-fields and render us confident that our choice of force-field yields an adequate description of the transition process.

## Materials and Methods

### MD Simulations

The starting structure for all simulations was the Hb T-state X-ray structure (Fermi et al. [24], PDB id: 2HHB). Each simulation was run for 200 ns with independent starting velocities. All simulations were carried out using the GROMACS 4.5.3 software [25], [26] with the GROMOS 43a2 force field [27] in explicit solvent and parameters as described before [7] with the exception of the Lennard-Jones cut-off (rvdw). Ten simulations used the same rvdw = 1.0 nm as Hub et al. Twenty additional simulations were run using a larger rvdw = 1.4 nm as consistent with the GROMOS force field parameterization. We did not observe differences in transition probabilities due to rvdw. Finally, 20 simulations using a second set of histidine protonations were performed (see Table 3). In total 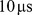 of Hb simulations were carried out.

To judge whether a T-R transition occurred in the individual simulations, the following criterion was applied: Each simulation was projected onto the difference vector between the T-state and the R-state X-ray structure (Park et al. [28], PDB id: 1IRD; with applied symmetry for the full tetrameric state). If a projection at any time covered 80% of the T-to-R distance, the whole simulation was considered a transition simulation. All transition trajectories covered a similar range on the T-R vector whereas one simulation exceeded that strongly. We excluded this outlier, because it would have dominated the global motions in terms of covariance despite its low statistical weight. This left us with 21 transition trajectories and 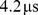 simulation time in total.

### Separation and Coupling of Quaternary and Tertiary Motions

The following method was applied to hemoglobin, but it can be used to analyse other systems with multiple domains as well. Here, we were interested in the coupling of inter- and intra-subunit motions and hence, we defined the protein chains to each be a local domain and focused on the chain-chain interfaces. Note that also other choices would have been possible, e.g. grouping Hb into two dimers instead of four monomers. When analysing the motions of a two domain protein consisting of only one chain, it may be of interest to consider each domain as one local entity.

### Tertiary-Only Motions: T

To obtain tertiary-only (T) coordinates, we superimposed the coordinates of each chain individually onto the respective chain in the T-state X-ray structure. This yielded an artificial trajectory with all chains having the same center-of-mass and orientation as in the T-state, not moving relative to each other and displaying only subunit-internal fluctuations. All four subunits were superimposed individually, reducing the degrees of freedom (DOF) of each by six (three translational and three rotational DOF) and resulting in 3N-24 DOF for the T trajectory (with N = 4556 being the number of non-hydrogen atoms).

### Quaternary-Only Motions: Q & cQ

The complementary quaternary-only (Q) coordinates were obtained by doing the opposite and superimposing the individual chains of the rigid T-state structure onto the respective chains of the MD structures, thereby leaving out the internal motions and only keeping the rigid body motions of the chains relative to each other. Thus each chain was represented by a rigid body (six DOF) and lost all information on internal coordinate changes. Since the six global DOF for the whole protein were removed, the Q motions sample 18 DOF. For the coupling analysis, we simplified the Q motions further using PCA. The eigenvalue spectrum shows a steep decrease among the 18 non-zero eigenvalues with the first eigenvector describing 22% of the total variance of the Q motions. This principal mode (referred to as cQ) was used for FMA.

### Application of FMA: cT & cTew

Functional Mode Analysis (FMA) identifies collective motions in a coordinate space (in our case T) maximally correlated to a specific functional property (in our case cQ). To avoid overfitting the high dimensionality of the T space needs to be reduced. The original implementation of FMA [14] accomplished a reduction of dimensionality through PCA on the underlying coordinates. Recently, a new version of FMA based on Partial Least Squares (PLS) was developed [15] where a linear model is constructed based on a given number of vectors in the coordinate space (so-called latent vectors) that are subsequently optimized. Thereby the number of latent vectors forming the subspace for the linear combination needs to be chosen correctly to avoid overfitting. We constructed the FMA model on the first half of the concatenated transition trajectories using the second half for cross-validation. 20 components were found optimal, with decreasing predictive power for a higher number of components (indicative of overfitting). We constructed the final model with 20 latent vectors on the full data set. The constructed model and the cross-validation are shown in Figure 2. The calculated Pearson correlation coefficients were 0.98 for fitting part and 0.83 for the cross-validation part indicating a strong coupling between motions along cQ and cT. For the further analyses of the interactions, the individual latent vectors underlying cT were reweighted with their contribution to the overall variance as described in [14] to yield the ensemble weighted coordinate cTew.

### Van der Waals Overlaps & Contact Analysis

For both analyses, we reassembled the coupled tertiary and quaternary motions in the following way: The MD trajectories were projected onto the plane spanned by cQ and cTew. The space between the minimal and maximal projections was divided equidistantly into 20 parts, yielding 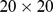 grid, covering all MD structures. Starting from the T-state we moved stepwise in this plane until each gridpoint was visited and mapped projections back onto the full structural space of the protein. This provided a subspace of 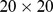 backprojected structures showing the coupling of local and global motions as derived from our simulations.

Note that one would expect a vdW overlap of zero for any full-dimensional MD structure. The fact that we did observe non-zero overlap values arises from the reduced dimensionality of the coupling plane: The vdW overlaps have been calculated for structures in the cQ-cTew hyperplane, which is a two-dimensional representation of the coupling process. Any such projection cannot fully cover the full-dimensional motion (and overlap) of atoms and therefore only shows the global trends. The fact that the maximal correlated coordinate from T (cTew) is reducing the vdW overlap when moving along cQ at the same time, points at (the prevention of) vdW overlaps as a global driving force for the coupling mechanism.

For each of these 400 structure the inter-chain vdW overlap was computed using a modified version of the dist program from the CONCOORD software [29]: For each atom we calculated how much its vdW sphere penetrates vdW spheres of atoms from other chains. The sum of vdW overlap values for all atoms gives a length in nm, representing a measure for the energetically unfavourable vdW overlap for each structure. For the contact analysis, we picked a specific pathway (referred to as cQ-cTew) in this hyperplane. Starting from the T-state, the first part of the path is along cQ, and the second along cTew For each of the 17 structures on this path, atoms closer to each other than 0.3 nm were monitored and defined a residue contacts. The calculation was carried out using g_contacts by Blau and Grubmüller [30].
